# Use of Biosensors as Alternatives to Current Regulatory Methods for Marine Biotoxins

**DOI:** 10.3390/s91109414

**Published:** 2009-11-24

**Authors:** Natalia Vilariño, Eva S. Fonfría, M. Carmen Louzao, Luis M. Botana

**Affiliations:** Departamento de Farmacología, Facultad de Veterinaria, Universidad de Santiago de Compostela, Campus Universitario, 27002 Lugo, Spain; E-Mails: natalia.vilarino@usc.es (N.V.); eva.fonfria@usc.es (E.S.F.); mcarmen.louzao@usc.es (M.C.L.)

**Keywords:** biosensor, seafood, shellfish poisoning, marine toxins, surface plasmon resonance

## Abstract

Marine toxins are currently monitored by means of a bioassay that requires the use of many mice, which poses a technical and ethical problem in many countries. With the exception of domoic acid, there is a legal requirement for the presence of other toxins (yessotoxin, saxitoxin and analogs, okadaic acid and analogs, pectenotoxins and azaspiracids) in seafood to be controlled by bioassay, but other toxins, such as palytoxin, cyclic imines, ciguatera and tetrodotoxin are potentially present in European food and there are no legal requirements or technical approaches available to identify their presence. The need for alternative methods to the bioassay is clearly important, and biosensors have become in recent years a feasible alternative to animal sacrifice. This review will discuss the advantages and disadvantages of using biosensors as alternatives to animal assays for marine toxins, with particular focus on surface plasmon resonance (SPR) technology.

## 1. Introduction

Marine biotoxins are compounds with toxic activity that accumulate in fish or shellfish and can cause human illnesses. They are usually produced by phytoplankton and reach shellfish or fish through the trophic chain. Toxic episodes occur as a result of the proliferation of some toxin-producing phytoplankton species or harmful algal blooms. The cause for this proliferation is unknown, in spite of an enormous increase in the frequency and worldwide distribution of the toxic bloom reports during the last decades [1], which probably also reflects an international improvement of monitoring programs [2].

The toxicity to humans has been usually reported as acute poisoning, and limits for the content of marine toxins present in seafood destined for human consumption have been set to protect human health. However, the possible chronic toxicity of a repeated exposure to sub-acute doses in humans is completely unknown for all the toxins. The impact of these toxic episodes reaches also ecological and economic levels. Marine wild animals that feed on contaminated species, such as marine mammals and birds, may present signs of intoxication and even die. The presence of a toxic bloom also generates important economic losses to the aquaculture sector and fish industry, as a consequence of the official regulations regarding toxin content in seafood and the bad publicity generated by human poisoning outbreaks [3,4].

Although marine toxins were initially classified according to the acute poisoning syndrome they induce in humans, nowadays a classification based on their chemical structure seems to be more widely accepted. In this review we will focus on the following groups of marine toxins: okadaic acid and derivatives, yessotoxins, pectenotoxins, azaspiracids, brevetoxins, cyclic imines, saxitoxin and derivatives, domoic acid, ciguatoxins and palytoxin and derivatives.

The implementation of appropriate regulatory limits for toxin contents in seafood destined to human consumption requires the availability of suitable detection methods, sensitive and reliable enough, to detect the presence of the toxins at the stated levels. Until very recently the regulations of most countries based the detection of marine toxins mainly on laboratory animal bioassays, except for the detection of domoic acid, which regulatory detection was by liquid chromatography high performance liquid chromatography with ultraviolet detection (HPLC-UVD) [2]. Therefore the detection of the regulated toxins, okadaic acid and derivatives, pectenotoxins, yessotoxins, saxitoxin and derivatives, and azaspiracids, has been done for decades by administration of the toxin to animals, in spite of the well known drawbacks of these techniques. Besides the ethical issues arising from the prolonged suffering and sacrifice of laboratory animals, these bioassays have also technical deficiencies, such as lack of sensitivity (the detection limits are often close to the regulatory limits), lack of specificity (the toxins cannot be identified and individually quantified), duration of the assay (too lengthy for the lipophilic toxins) and too high a rate of false positives and negatives [5-7].

Although the desire to move away from animal bioassays has been recognized in the legislation of many countries, such as in the European Directive 86/609/EEC [8], for a variety of alternative detection methods that have been developed during the last decades the criteria for substitution set by regulatory authorities are difficult to meet. These rigid criteria are aimed to guarantee consumer protection and, unless there is solid evidence of an adequate level of protection by internationally validated methods, regulatory authorities are reluctant to accept replacements. Currently, the detection of saxitoxin and analogs by high performance liquid chromatography with fluorimetric detection (HPLC-FLD) is officially accepted in some countries [9,10] and an ELISA (enzyme-linked immunosorbent assay) has also been published as an AOAC method for the detection of domoic acid [11].

In addition to the officially accepted methods, there is a wide variety of techniques that have been developed for the detection of marine toxins. Among them there are several analytical methods such as high performance liquid chromatography (HPLC) with UV or fluorimetric detection, liquid chromatography-mass spectrometry (LC-MS), and liquid chromatography-tandem mass spectrometry (LC-MS/MS), that have been optimized for many toxin groups and have become very popular in recent years. These analytical methods allow the unequivocal identification of the toxins present in a sample and their quantification with high levels of sensitivity. However, they require specialized laboratory personnel and expensive equipment. A major disadvantage of these methods is the need of certified standards for each known analogue of every group of toxins in order to evaluate total toxicity, and in any case the contribution of unknown compounds to sample toxicity will not be considered. The lack of standards is an important obstacle for the substitution of the mouse bioassay by other analytical methods. Another group of alternative techniques are biological methods that have also been developed for most of the toxin groups with a great variety of designs, from cytotoxicity assays to biosensor techniques. These methods vary in specificity, but none of them can identify the different analogues of a toxin group. Their practicality depends on the technological approach, as well as their cost. Biosensor technologies can offer cost-effective solutions for marine toxin detection with suitable characteristics of group specificity, sensitivity, portability, repeatability and robustness. One of the problems of the present situation regarding the analysis of marine toxins is the lack of uniformity in the analytical outcomes by different laboratories. The characteristics of biosensors would support their use, not only to obtain more uniform results, but also to comply with the rapidly increasing demands of certification and traceability of traded seafood.

Biosensor-based technologies have been widely used in the last 15 years for pharmacological, environmental and food safety applications [12-14]. A biosensor is an analytical device incorporating a biorecognition element intimately associated with or integrated within a transducer that converts the biological response into an electrical signal. A great variety of detection techniques can be included in this definition. The biological response could be anything from enzyme activity or antibody/receptor binding to cell responses. The transduction to an electrical signal could also be diverse. Biosensor technologies include transduction platforms based on electrochemical (potentiometric, amperometric, impedance), piezoelectric, thermal or optical methods (reflectrometric interference spectroscopy, interferometry, optical waveguide lightmode spectroscopy, total internal reflection fluorescence, surface plasmon resonance…) [14,15]. These techniques have been adapted to detect analytes of interest based on the interaction with or functionality modification of a biological target, which could be nucleic acids, enzymes, antibodies, receptors, cell organelles or whole cells [12-16]. The specificity of the detection is determined by the biological component of the method. For example, a method based on binding to a specific antibody would be very specific, however whole cell-based biosensors usually lack that degree of specificity and that characteristic could be used as an advantage in a broad-spectrum detection/monitoring technique. The sensitivity, on the other hand, as well as the portability of the device, depends on the signal transducer. In recent years the microfabrication tools have made possible the idea of microbiosensors or nanobiosensors, a very young branch of biosensors with a technologically challenging future [13]. However the statistical significance of single molecule/cell detection should be addressed before extended use of these techniques. Biosensor assays may have mainly two designs, a direct or an indirect format [13]. The direct format is based on the detection of analyte binding to a target or being cleaved by an enzyme, for example. In the indirect format an additional reaction has to occur in order to detect the analyte, for example the analyte may inhibit the interaction of the biological target with a “reporting element”. Indirect assays are often used in food analysis because they usually display lower interferences with complex matrixes. Actually, sample preparation is commonly a critical step in method development when working with food samples due to their complexity, and seafood is not an exception. Therefore, attention should be paid not only to the efficiency and sensitivity of the biosensor assay but also to the sample preparation procedure. This review will revise the biosensor techniques that have been developed for the detection of the different groups of marine biotoxins. Although the main focus will be on biosensor devices, other techniques that use biological responses for toxin detection will be also included, since their scientific approaches and technical developments may serve as basis for future biosensor designs in seafood toxin detection.

## 2. Okadaic Acid and Derivatives

**Figure 1.**
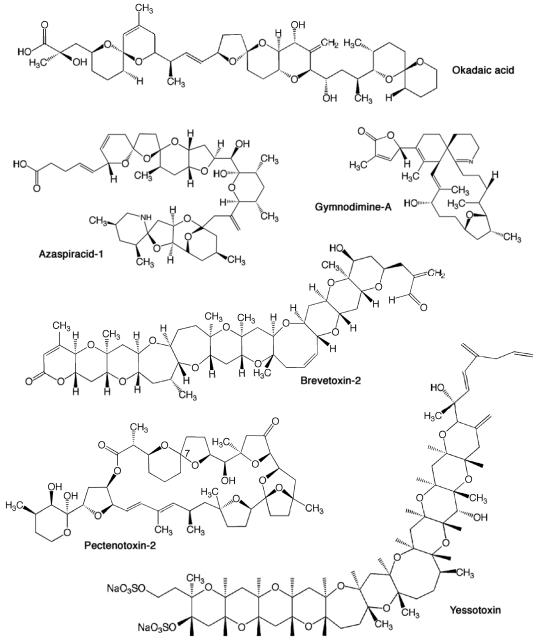
Chemical structures of lipophilic toxins.

Okadaic acid (Figure 1) and its derivatives are the causative agents of the so-called diarrheic shellfish poisoning (DSP). These toxins have a worldwide distribution with a higher occurrence in Europe and Japan [17]. The group includes okadaic acid and dinophysistoxins, which are produced by microalgae of the genus *Dinophysis* and *Prorocentrum* [18-20]. These compounds are well known inhibitors of protein phosphatases, mainly PP2A and PP1 [21,22]. DSP is produced by acute intoxication after consumption of contaminated mollusks and includes gastrointestinal symptoms, such as nausea, vomiting and diarrhea, and headaches [23]. Although no fatalities have been reported, these toxic episodes cause high economic losses, both in the health care and aquaculture sectors. The presence of DSP toxins in shellfish destined for human consumption is legislated in many countries. In most countries the regulatory limit for okadaic acid and derivatives and pectenotoxin, based on the acute intoxication data, is 0.16 mg of okadaic acid equivalents per kg of shellfish meat (whole body or any edible part) and the official detection method is the mouse bioassay [2,24]. The European regulation contemplates alternative detection methods for lipophilic toxins including HPLC with fluorescence detection, liquid chromatography-mass spectrometry, immunoassays and functional assays such as the phosphatase inhibition assay [25], however the lack of validation studies and standards for all the required analogues of each group have precluded their implementation as alternatives to the mouse bioassay in the EU. The chronic toxicity of these toxins to humans is unknown, but the tumour promoting effects observed in animals [26,27] have raised concerns about the current safety limits both in the scientific and health care communities.

Several immunosensors have been developed for the detection of okadaic acid and its derivatives. Different transduction technologies were adapted for the immunodetection of okadaic acid, including quartz crystal microbalance [28], chemiluminiscence integrated into a flow injection analysis system [29], surface plasmon resonance [30,31] and electrochemical methods [32-34]. All these DSP-immunobiosensors are designed as competition assays with enough sensitivity to detect okadaic acid at the concentrations required by the current legislation. However, the recently published, enzymatic recycling system for signal amplification coupled to an amperometric immunosensor displays a substantial improvement in sensitivity [32]. The problem of most immunosensors is the lack of correlation between the cross-reactivity of the antibody with each toxin analog and the toxic potency *in vivo*. This problem has been overcome for okadaic acid, dinophysistoxin-1 and dinophysistoxin-2 with the development of a monoclonal antibody used in a SPR-based biosensor, which cross-reactivity towards these three compounds matches their toxic potency both in buffer and shellfish extract [31].

DSP toxin biosensors have also been developed using a well known target of these group of toxins, the protein phosphatase PP2A [35,36]. The inhibition of the enzymatic activity of PP2A is measured by electrochemical detection, either by direct immobilization of the enzyme on a screen-printed electrode [35] or using a bienzyme amplification system with off-line enzymatic incubation with PP2A [36]. Although the second approach offers a higher sensitivity, the first enzyme biosensor has also enough sensitivity to detect okadaic acid contents below the limit established by the legislation, however their performance with shellfish matrixes has not been tested yet.

Intense work in the development of ELISAs [32,37] and phosphatase inhibition assays [38-41], for the detection of DSP toxins, has been the basis for the wide array of biosensor technologies designed for the detection of these compounds. Some detection methods based on the *in vitro* cytotoxicity of DSP toxins have been also described [42-44], and although they have several drawbacks, such as practicality of use and lack of specificity for the identification of toxins, they might be the basis for future developments of universal cell-based detectors for marine toxins. The analytical methods that can be used to detect these toxins include LC-MS and HPLC-FLD [45-49].

## 3. Pectenotoxins

Pectenotoxins are macrocyclic polytethers (Figure 1) that accumulate in filter-feeding shellfish. They are produced by microalgae of the genus *Dinophysis* and pectenotoxin-contaminated shellfish has been reported from countries all over the world [50]. About 13 pectenotoxins have been described as natural compounds present in shellfish, with structural variations that determine important variations in toxicity [51]. The parental phytoplanktonic compounds display a higher toxicity that is reduced after transformation by shellfish. Although these toxins were initially classified in the DSP group, their diarrheic toxicity to humans has not been proven. Actually, it has been recently demonstrated that oral administration to mice does not cause any toxic effect, in spite of the toxicity induced by intraperitoneal administration [52,53]. The mechanism of action of pectenotoxin seems related to the disruption of the actin cytoskeleton observed *in vitro* [54-57]. The presence of pectenotoxins in seafood is regulated in several countries. In the EU, as well as in New Zealand and Chile [2], the content of okadaic acid, dinophysistoxins and pectenotoxins must not exceed 0.16 mg of okadaic acid equivalents/kg of shellfish meat [24].

Besides the mouse bioassay there are not many methods available for the detection of pectenotoxins that use biological components. To our knowledge no anti-pectenotoxin antibodies have been reported so far, probably due to the small amount of pure pectenotoxins available worldwide. Functional, multi-toxin detection assays have been developed based on the induction in hepatocytes of apoptosis or cytotoxicity by several toxins, including pectonotoxins [58], but this cell based-assay is not specific for pectenotoxins and although its performance with shellfish extracts was tested, it is not very practical since apoptosis is judged by microscopy. Another multitoxin detection assay based on cytotoxicity in differenct cell models has been described recently that includes the detection of pectenotoxin with a more practical approach [42]. However, as we said above the field of cell-based biosensors is growing fast and their use as universal toxin detectors may hold interesting options for marine toxin detection in the future. These toxins can also be detected by the chemical detection methods HPLC-UVD, HPLC-FLD and LC-MS [18,47-49,59,60].

## 4. Yessotoxins

Yessotoxins are a group of marine toxins with a ladder shape polycyclic ether structure (Figure 1). There are more than 36 compounds in this group of toxins [61]. Like other groups of marine toxins, yessotoxins have phytoplanktonic origin (*Protoceratium reticulatum, Lingulodinium polyedrum, Gonyaulax spinifera*) [62-64] and a worldwide distribution [61,65]. Although yessotoxins were initially included in the DSP toxin group, no diarrheic effects have been reported in humans and they have been demonstrated to have no diarrheic effects in mice by oral administration [66,67]. However they display a high intraperitoneal toxicity in the mouse bioassay [66,68]. Their mechanism of action is still unclear. Several *in vitro* cellular effects have been reported, but their correlation to *in vivo* toxicity of yessotoxin, which is considered a cardiotoxic compound, remains to be elucidated. The presence of yessotoxins in shellfish is regulated in Europe and other countries [2]. The regulatory limit is 1 mg of yessotoxin equivalents per Kg of shellfish meat (whole body or any edible part) [24].

Several biosensor-based techniques have been developed for the detection of yessotoxin. Surface plasmon resonance and resonant mirror biosensors have been used for its detection based on its interaction with phosphodiesterase enzymes, using either direct format or competition format assays [69-72]. The interaction with phosphodiesterase allows the detection of yessotoxin in the low μM range, enough for current regulatory limits, however the method is not as specific as immuno-based biosensors, since other compounds such as the brevetoxin PbTx1 and other polyethers can also interact with phosphodiesterases [72]. Other methods with biological components include a microplate assay based on the activation of phosphodiesterase enzymatic activity by yessotoxins [73], which was the basis for the development of these biosensor methods, and a direct assay that detects the interaction of these toxins with phosphodiesterases by fluorescence polarization [74]. Another functional method for the detection of yessotoxin involves measurement of the E-cadherin fragment ECRA100 and total E-cadherin by protein blot [75,76]. Although sample processing was shortened recently, this technique takes much longer than the previously mentioned methods, since a 20 h incubation of the cells in the presence of toxin is required. Additionally, this method is not specific for yessotoxins because the same fragment of E-cadherin can be detected after azaspiracid treatment, and both toxins have similar potency and efficacy [77]. ELISAs are also available for the detection of yessotoxins [78,79]. ELISAs are fast, sensitive and specific and can be used as screening assays for high numbers of samples. However, the cross-reactivity of the antibodies with the different yessotoxins does not match their relative toxic potencies, and the antibodies used in these assays are polyclonal and therefore their stock is limited. Additionally, there is an overestimation of toxin content versus LC-MS, which could be expected given the fact that quantification by LC-MS was done only for three toxins of this group, but no comparison to mouse bioassay results was done. As mentioned a few lines above, the detection of yessotoxin is also possible by LC-MS, besides the other analytical methods HPLC-FLD and capillary electrophoresis [47-49,80-82].

## 5. Azaspiracids

The azaspiracids were described for the first time in 1995 [83]. This group of toxins comprises more than 20 analogs (the azaspiracid-1 structure is given in Figure 1) [84-88]. They are produced by phytoplankton (*Protoperidinium crassipes*) and accumulate in shellfish through the trophic chain [89]. Some compounds have been isolated only from shellfish and they are thought to be the product of metabolization of the parent phytoplanktonic compounds. The presence of these toxins has been described in shellfish from Ireland, United Kingdom, Spain, France, Norway and Morocco [83,90-93], among others. Human intoxication by azaspiracid-contaminated shellfish (azaspiracid poisoning, AZP) has been reported and includes nausea, diarrhea and headache symptoms, similar to DSP. The content of azaspiracids in shellfish destined to human consumption is regulated in many countries; in the EU the regulatory limit is 160 μg of azaspiracid equivalents/kg of shellfish meat (whole body or any edible part) [24]. The European official method for the detection of azaspiracids is the mouse bioassay [25].

Currently, there is no biosensor assay for the detection of azaspiracids, and besides the mouse bioassay, there are only two other options for the detection of these toxins. LC-MS techniques have been optimized for the detection of azaspiracids [48,49], although quantification and identification is possible only for those compounds that have available certified standards. Azaspiracid-specific antibodies have been produced recently, and shown to bind several azaspiracid analogs (Aza-1, 2, 3 and 6) [94,95]. Competition and sandwich ELISAs were designed with these antibodies and a synthetic fragment of the azaspiracid molecule that is conserved for many analogs. Hopefully, in the near future these new antibodies will be available for the detection of azaspiracids in electrochemical or optical immunosensors.

## 6. Brevetoxins

Brevetoxins (PbTx, Figure 1) are lipophilic polyether toxins responsible for neurotoxic shellfish poisoning (NSP). They are classified into two types, depending on their backbone structure: type A (PbTx-1, 7, 10) and type B (PbTx-2, 3, 5, 6, 8, 9, 11, 12, 13, 14) [96]. Globally, they occur mainly in Mexico, USA and New Zealand and they are produced by the species *Karenia brevis* [97,98]. Brevetoxins bind with high affinity (K_D_ 1–50 nM) to site 5 of the voltage-dependent sodium channel α-subunit [99] resulting in a sustained sodium influx and consequent depolarisation of neural membranes. This property causes their toxic effects in humans, which include gastrointestinal and neurologic symptoms such as nausea, vomiting, diarrhoea, chills, hot-cold flashes, hypotension, cramps, arrhytmias, paraesthesia, motor incoordination, double vision, bronchoconstriction and paralysis [65,100]. Although brevetoxins have been related to the death of fish, birds and some marine mammals [101-103], no human mortalities associated with these toxins have been reported to date. Currently, the accepted detection method for brevetoxins is the mouse bioassay with diethyl ether extraction of shellfish tissue based on the American Public Health Association (APHA) method [104]. Basically, any detectable level of brevetoxins per 100 g of shellfish tissue was considered potentially unsafe for human consumption. In practice, a residue toxicity ≥ 20 MU (mouse unit: amount of crude toxic residue that will kill 50 percent of the test animals in 930 minutes [105]) per 100 g shellfish tissue was adopted as the guidance level for closure of shellfish harvesting areas in USA, Mexico and New Zealand [2,65].

Apart from mamamalian bioassays, several methods have been developed to detect brevetoxins. In the biosensors field, a surface plasmon resonance-based detection method for ladder-shaped polyeher compounds (among them brevetoxin-2) has been recently published [72]. The ability of these molecules to inhibit the interaction of desulfo-yessotoxin to phosphodiesterase II was used to design an indirect assay format that can detect several toxins. In the case of PbTx-2, inhibition was achieved in the μM range. However, this assay has not been tested in shellfish matrixes and the data point to a lack of specificity since toxins from different groups can be detected, including yessotoxins. Besides this SPR technique, there is an immunosensor with amperometric detection [33] that allows the detection of PbTx-3 in the ng/mL range. Finally, a neuronal network biosensor has been also developed for the detection of the neurotoxins PbTx-3 and saxitoxin in buffer and diluted seawater [106]. The main feature of this method is its high sensitivity with detection limits of 296 pg/mL and 12 pg/mL for PbTx-3 and saxitoxin, respectively, although it lacks specificity.

Other detection methods that use biological components for the detection of brevetoxins include receptor binding assays [107], radioimmunoassay [108,109], ELISA [79,110,111] and fluorimetric assays based on changes in membrane potential [112]. The analytical methods that have been used to detect this group of toxins are LC-MS [113] and micellar electrokinetic capillary chromatography (MEKC) coupled to laser-induced fluorescence detection [114].

## 7. Cyclic Imines

The cyclic imines phycotoxins are a heterogenous emerging group of marine compounds that includes the gymnodimines (Figure 1), spirolides, pinnatoxins, pteriatoxins, prorocentrolides and spiroprorocentrimines [115]. All of them share an imine group as a part of a cyclic ring system within their molecular framework. They are produced by microalgae of species that differ among the cyclic imine subgroups. Although their mechanism of action is not fully understood, it has been demonstrated that gymnodimines and spirolides target the nicotinic acetylcholine receptors [116-118]. The cyclic imines display a “fast acting toxicity” with an acute threshold response (“all or nothing”) in mammalian bioassays [119]. At lethal concentrations they cause death in a few minutes after intraperitoneal injection, while at sublethal doses mice recover rapidly. Their toxic potential is much lower via the oral route [120]. At present, no human intoxication has been unequivocally linked to these toxins and they are not still regulated, although they may pose a real threat to human health. Moreover, due to their high toxicity by intraperitoneal administration, the cyclic imines are a source of false positives in DSP and NSP toxin detection by the mouse bioassay. Several detection methods for these toxins have been developed while the scientific community works to provide more toxicological data for a more informed evaluation of the human health threat related to these toxins.

Until recently, the cyclic imines could only be detected by mouse bioassay and LC-MS-based detection techniques [48,121-125]. The only method currently available that has a biological component, besides the mouse bioassay, is a fluorescence polarization assay for the detection of gymnodimines and spirolides [126]. A competition assay format uses the ability of these compounds to inhibit the interaction between nicotinic acetylcholine receptors and α-bungarotoxin. This method allows the quantification of gymnodimine-A and 13-desmethyl C spirolide in the nM range. This assay is sensitive enough to detect concentrations of these toxins higher than 85 μg/kg in shellfish meat using an acetone/chloroform extraction with acceptable recovery rates. The use of a biological target and its well known specific interaction with α-bungarotoxin, avoids the interference of other phycotoxins, ensuring its specificity [126].

## 8. Saxitoxin and Analogues

**Figure 2.**
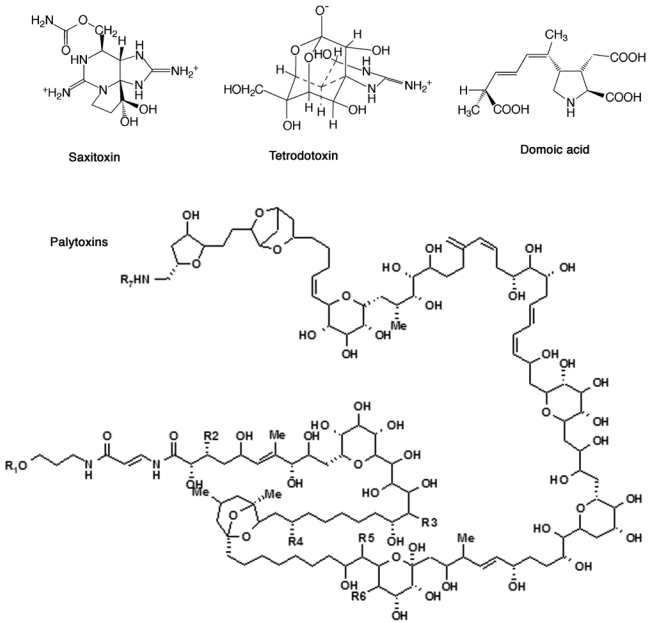
Chemical structures of hydrophilic toxins.

This phycotoxin group comprises saxitoxin (STX, Figure 2) and its analogues, more than 24 potent water-soluble neurotoxins that differ in combinations of hydroxyl and sulphate substitutions located at four sites of a tetrahydropurine backbone. Based on substitutions at R_4_, the saxitoxins can be subdivided into four groups: the carbamate, sufocarbamoyl, decarbamoyl and deoxydecarbamoyl toxins [99]. These toxins are responsible for paralytic shellfish poisoning (PSP), the most widespread algal-derived shellfish poisoning worldwide. On a global basis, almost 2,000 cases of human intoxications are reported per year, with a 15 % mortality rate [127]. STX and analogues are produced by the genus *Alexandrium, Gymnodinium* and *Pyrodinium* and elicit their effects by binding with high affinity to site 1 of the voltage-dependent sodium channel α-subunit [128,129] and blocking the sodium influx that prevents the generation and propagation of action potentials in excitable cells [130,131]. This molecular effect causes both neuronal and gastrointestinal symptoms in humans; such as numbness, headache, dizziness, nausea, vomiting, diarrhea, parestesia, paralysis, hypotension, respiratory difficulty and in extreme cases death [132]. Currently, in the European Union and in most of American and Asiatic countries a regulatory limit of 800 μg of saxitoxin equivalents/kg of any edible part of molluscs has been established [2,24], being the mouse bioassay the official method for their detection [25]. Recently, a new detection method has been validated in North American and European countries, a high performance liquid chromatography method with fluorescence detection (HPLC-FLD), the so-called Lawrence method [10,133].

Several biosensor techniques have been adapted to the detection of PSP toxins. Chemosensors based on the photoinduced electron transfer (PET) principle sense toxins by means of synthetic fluorophores, since the toxin binding produces a fluorescence enhancement. Coumaryl [134], anthracylmethyl [135], acridinylmethyl [136] and boron azadipyrrin [137] crown ethers have been synthesised and evaluated as fluorescence recognition molecules for STX. Among them, boron azadipyrrin crowns, with a binding constant for STX in the μM range, allowed working in the visible region of spectrum, far from any shellfish matrix absorption bands. The detection limit of the mouse bioassay used for PSP toxins determination corresponds approximately to 1 μM of STX, and the detection limit of this chemosensor assay is slightly below it [137]. New fluorophores are being investigated to improve its performance. A surface plasmon resonance-based assay for the detection of saxitoxin and analogues has also been recently published [138,139]. An inhibition format was designed using a STX-chip and monoclonal or polyclonal antibodies. This method allows the quantification of several STX analogues (STX, dcSTX, C1/2, GTX2/3, dcGTX2/3 and GTX5) at concentrations five times lower than the regulatory limit in extracts of several shellfish matrixes. In spite of the low cross-reactivity of the antibodies with toxins hydroxylated at the site R1, which is a common feature of the antibodies developed against PSP toxins [140,141], its performance with natural shellfish samples (in relation to mouse bioassay and HPLC-FLD method) supports its use as a screening method for saxitoxin and analogues detection. Besides this two methods, some other biosensor assays based on functional responses that can detect PSP toxins are being discussed in the tetrodotoxin section, since they where initially developed for this other toxin that has the same mechanism of action as PSP toxins. Also, the neuronal network biosensor described in the brevetoxin section has been reported to detect saxitoxin with high sensitivity [106].

In addition to these techniques, saxitoxins and analogues can be detected by radioimmunoassay [141], receptor binding assays [142-144], electrophysiological assays [145], fluorimetric assays based on changes in membrane potential [146,147] and ELISA [79,140], all of them based on biological detection and the analytical technique capillary electrophoresis [148], among others.

## 9. Domoic Acid Group

This phycotoxin group comprises ten potent water-soluble neurotoxins, domoic acid (DA, Figure 2) and its isomers, which are responsible for amnesic shellfish poisoning (ASP) [149]. This group of toxins produced by the genus *Pseudonitzschia* and *Nitzschia* and *Chondria armata* has a worldwide distribution [150-153]. The mechanism of action of these compounds involves their interaction with kainate receptors (K_D_ 5 nM [99]), a subclass of glutamate receptors, and their activation. ASP symptoms in humans include nausea, vomiting, diarrhea, abdominal cramps, dizziness, headaches, disorientation, memory loss, seizures, coma and death in extreme cases [154]. In the European Union, the regulatory limit for the total ASP toxin content in the edible parts of molluscs is 20 mg/kg [24], being the reference method to detect these toxins the HPLC-UVD [25]. The mouse bioassay is not useful to detect domoic acid in shellfish since the detection limit of this technique for ASP toxins (400 mg/kg) is higher than the regulatory limit. Recently, an ELISA has been published as AOAC Method for the detection of DA in shellfish and approved as official detection method in many countries [11,155].

Several immunosensors have been designed for the detection of domoic acid with different transducer technologies. For surface plasmon resonance technology, the toxin was covalently linked to a gold-coated chip with mixed oligo ethylene glycol self-assembled monolayers [156], and subsequently the DA in solution was detected by competition with the immobilized toxin for binding to an anti-DA monoclonal antibody. The detection limit was 0.1 ng/mL DA, showing better results than the ELISA performed using the same antibody [157]. A second SPR-based assay for the detection of DA was developed using a chip with DA immobilized on its surface and a rabbit polyclonal antibody. A simple extraction with methanol allows the detection of DA in the range of ng/g, in scallops, cockles, mussels and oysters [158]. Also using a surface plasmon resonance-based transducer, another DA detection method combines recognition elements based on ultra-thin molecularly imprinted polymer (MIP) films [159]. Although its sensitivity is still low, these synthetic receptors are promising since their stability and performance in SPR instruments are better than for natural targets. Two electrochemical-based immunosensors have been developed using screen-printed carbon electrodes coupled to amperometric [33] or differential pulse voltametry detection [160]. In both cases, the presence of DA is detected by competition with immobilized DA for binding to an anti-DA antibody and the signal is generated by an electroactive product of alkaline phosphatase. The detection limits of these techniques are similar: 2 ng/mL and 5 ng/mL, respectively, but differ in the analysis time (30 min and 150 min respectively). Both methods would allow the on-site measurement of DA. These techiques are useful for the detection in mussel matrix with good performance and good recovery rates (higher than 83 %). Domoic acid can also be detected by LC-MS [47,161], thin-layer chromatography (TLC) [162] and capillary electrophoresis (CE) [163].

## 10. Ciguatoxins

Ciguatoxins (CTXs) are a family of lipid-soluble highly oxygenated cyclic polyether compounds which act by binding to site 5 of the voltage-dependent sodium channel α-subunit, a site overlapping the brevetoxin binding site [65,164,165]. These toxins are the main agents responsible for ciguatera fish poisoning (CFP), a seafood intoxication caused by ingestion of some species of tropical and subtropical reef fish. CFP presents neurological (paresthesia, dizziness, headache, numbness, ataxia, coma and death), gastrointestinal (diarrhea, nausea, vomiting and abdominal pain) and cardiac symptoms (bradycardia and hypotension) [99]. Although it is rarely fatal, CFP affects more than 50,000 people annually [99]. Very few specific regulations exist for ciguatera toxins [166]. In the European Union, Regulation (EC) No 853/2004 establishes that fishery products containing biotoxins such as ciguatoxins must not be marketed but no information about analytical methods is given [24]. Traditionally, the mouse bioassay based on the method described by Banner *et al.* has been used to detect CTXs in contaminated fish [65] but its lack of specificity and other associated problems encouraged the development of alternative methods.

No biosensor-based method is available for the detection of CTXs. A recent publication of a surface plasmon resonance-based detection method for ladder-shaped polyether compounds similar to CTXs [72] may offer a possibility for the detection of this class of toxins by biosensors, although CTX was not tested in this assay and the sensitivity of the method for the other toxins suggests a difficulty to comply with the low CTX detection limits required to ensure human safety.

Other methods that use biological components have been developed for the detection of CTXs, such as RIA [167], ELISA [168-171], rapid enzyme immunoassay stick test [172,173] or fluorimetric assays based on changes in membrane potential [112]. CTXs can also be detected by LC-MS [174].

## 11. Palytoxins and Ostreocins

Palytoxin (Figure 2) is a more complex and one of the more potent marine biotoxins [175]. Palytoxin and its analogs were initially found in marine zoanthid corals and sponges and also in some species of dinoflagellates, such as *Ostreopsis siamensis* and *Ostreopsis ovata*, but it has been also described in fish and shellfish [176]. The hypothesis of a symbiotic bacterial origin for this group of toxins has been explored in some recent works [177,178]. Although the palytoxin-containing zoanthid organisms are located in tropical waters, the dinoflagellates of the genus *Ostreopsis* have a worldwide distribution and toxin producing species have been described not only in tropical and subtropical waters, but also in the Mediterranean Sea [179-182]. Humans show symptoms of intoxication by palytoxin following ingestion of contaminated seafood or exposure to seawater aerosol in bathing areas. Oral poisoning can induce among other symptom vomiting, diarrhea, paresthesia of the extremities, myalgia, repiratory distress and death [183-185], while exposure to the toxin by aerosol induces rhinorrhea, cough, fever and broncoconstriction [186]. The mecanism of action of this toxin, at least for its *in vitro* effect on cells, involves binding to the Na^+^/K^+^ ATPase and converting the functionality of the pumps into non-selective ion channels which alters the membrane potential [187-190]. The presence of palytoxin in seafood destined to human consumption is not regulated, but its high intraperitoneal toxicity and the reported toxic episodes in humans suggest the usefulness of detection methods.

Several enzyme-linked immunosorbent assays have been developed for the detection of palytoxin with sensitivities in the low ng/mL range [177,191]. These ELISA assays can detect different palytoxins, but also non-toxic palytoxin derived compounds [191,192]. There are also several cell-based assays where hemolytic or cytotoxic activity of palytoxin is identified by neutralization with specific antibodies or ouabain [190,193]. However, these technologies have not been transferred to biosensor devices with integrated biological response and transduction detector.

## 12. Tetrodotoxin

Tetrodotoxin (Figure 2) has been found in several species of tropical fish, gastropod mollusks, crabs and newts [194-197]. Although tetrodotoxin-contaminated fish, such as puffer fish, are found in tropical waters all over the world, poisoning episodes have been mainly described in Japan and other Asiatic countries [196,198], as the species that accumulate the toxin are not part of the diet in other parts of the globe, and therefore only sporadic poisonings have been described in Mexico, USA and Europe. This toxin of bacterial origin induces neurotoxic symptoms similar to the saxitoxin group. In spite of their different structures tetrodotoxin and saxitoxin are inhibitors of voltage gated Na^+^ channels, binding to site 1 of the α-subunit [199]. The symptoms caused by tetrodotoxin poisoning include parestesias of several areas, nausea, vomiting, motor paralysis, incoordination, and even death by respiratory arrest [195,198]. The importation of puffer fish and other toxic species is not allowed in many countries, including the USA and Europe, and therefore no regulatory limits have been established [100,200]. The commercialization of certain tetrodotoxin-toxic species has been restricted in Asiatic countries [100].

The detection of tetrodotoxin, one of the most dangerous sea-born toxins, is essential for human health preservation in some countries. Several biosensor techniques have been developed that allow the detection of tetrodotoxin. One of the approaches uses an anti-tetrodotoxin specific antibody as the biological reporter and amperometric detection with a screen-printed electrode [33]. The format of the assay is an indirect competition assay where the amount of current generated by *p*-aminophenol, the product of the enzymatic activity of the alkaline phosphatase label of the specific antibody, is inhibited by the presence of tetrodotoxin in a sample that competes with the electrode immobilized tetrodotoxin. Another electrochemical biosensor technique for tetrodotoxin is based on differential pulse voltametry also with screen-prinded electrodes to measure the *p*-aminophenol product of alkaline phoshatase activity [201]. The design of this assay is also a competition immunoassay, but in this case the molecule labeled with the alkaline phosphatase is tetrodotoxin.

A tissue biosensor has been also developed with frog bladder membranes, which have a high concentration of Na^+^ channels [202-204]. A Na^+^ specific electrode measures the transport of Na^+^ through the membrane and its dose-dependent inhibition by tetrodotoxin. Another tetrodotoxin biosensor based on inhibition of cell function has been designed using murine spinal cord neuronal networks cultured on microelectrode arrays [205]. The biological response is monitored as extracellular potentials. In this system, tetrodotoxin quantification was based on spike rate inhibition.

The performance of these biosensor assays with shellfish extracts has been reported only for the frog bladder biosensor. The neuronal network biosensor and the electrochemical immunosensors offer the advantage of portability. However, the portability of the biosensor does not ensure that in-field measurements can be performed if a simple enough sample preparation technique is not optimized for that purpose. An advantage of the biosensors based on functional responses is that they can be used to detect more than one compound with similar mechanisms of action, and usually the sensitivity is correlated with toxic potency. The frog bladder sensor has been demonstrated to be sensitive also for PSP toxins and it would be expected that the neuronal network biosensor could also detect this group of toxins. Actually, neuron-based biosensors could be used to detect many neurotoxins, acting as general detectors [206]. However, this promising technology will have to resolve some technical issues, such as the maintenance of neuron network cultures with all its logistic and economic problems.

All these techniques have a lower detection limit than the mouse bioassay for tetrodotoxin. However, as we said before, the performance of some of these biosensor assays with sample extracts is still to be tested.

Several ELISA assays have been developed as well for the detection of this toxin [33,201,207,208] some of them preparing the ground for the development of the electrochemical immunosensors mentioned above. A neuroblastoma culture assay in a microplate and a rapid hemolysis assay are also available for the detection of tetrodotoxin based on the inhibition of veratridine/ouabain-induced cell death [209]. Another recent technology that could be adapted in the future to detect tetrodotoxin and other marine neurotoxins using biosensors is the incorporation in lipid bilayers of recombinant human voltage-gated sodium channels [210].

## 13. Concluding Remarks

There have been great advances in the use of biosensors for marine toxin detection and the future is still more promising. The current situation is that for most groups of toxins there are biosensor technologies with enough sensitivity to comply with the regulatory limits. However, none of these methods has been validated and/or accepted as an alternative to the mouse bioassay. Actually, in most cases these techniques would be good tools to be used at least as screening methods in order to reduce the number of animal bioassays.

In general, biosensor technologies have some advantages versus analytical methods and animal bioassays that include low cost, ease-of-use, speed, no need of highly trained lab personnel and automation, most of them with very good reproducibility and robustness. Moreover, these methods do not entail legal or ethical issues related to the use of laboratory animals. The evaluation of the toxicity of a sample with biosensor-based techniques does not require the use of a toxin standard of every compound of a toxin group, just a representative member would suffice, which is one of the more important drawbacks of analytical methods, since certified standards for many marine toxin analogues are not available or easy to produce.

In the field of immunosensors, it is important to keep in mind that the ability of the antibodies to detect the different members of a toxin group is based in the immune response of a host to an antigen (usually a protein-coupled toxin), which is not related to the toxic potency of these compounds. Antibodies with a good correlation of cross-reactivity and toxic potency are rarely obtained, and when it happens it is mainly for toxin groups with a reduced number of analogues. As a consequence, the quantification of the toxin content of a sample by antibody-based methods does not often reflect accurately its toxicity. Sometimes the results of immunoassays are reported as equivalents of a representative toxin of the group, in the same way the regulatory limit is established, however the regulatory limit refers to toxic equivalence, so the use of that terminology in immunoassays/sensors is misleading. In spite of these disadvantages, these assays are sensitive, reliable, robust, easy to perform, portable techniques and therefore worthy alternatives for screening purposes. Additionally, the search for new, improved antibodies has already produced detection tools with a very good correlation between toxicity and cross-reactivity, such as in the case of okadaic acid immunosensors [31].

Functional and biological receptor-based assays/sensors usually provide a better evaluation of sample toxicity, since the measurement is based on the mechanism of action of the toxin. However, the robustness and portability of functional/receptor-based techniques is not as good as for immunosensors, because receptors and cells are usually more labile than antibodies. Some practical issues have to be overcome before an extended use of cell-based sensors such as the need to maintain cell cultures. The use of receptors in an extended technique for the detection of some groups of toxins is really feasible these days. For example, the reagents needed for the cyclic imine receptor-based assay have good stability in laboratory storage conditions [126].
